# PFK15, a Small Molecule Inhibitor of PFKFB3, Induces Cell Cycle Arrest, Apoptosis and Inhibits Invasion in Gastric Cancer

**DOI:** 10.1371/journal.pone.0163768

**Published:** 2016-09-26

**Authors:** Wei Zhu, Liang Ye, Jianzhao Zhang, Pengfei Yu, Hongbo Wang, Zuguang Ye, Jingwei Tian

**Affiliations:** 1 School of Life Science and Bio-pharmaceutics, Shenyang Pharmaceutical University, Shenyang, Liaoning 110016, China; 2 State Key Laboratory of Long-acting and Targeting Drug Delivery System, Non-clinical Research Department, Luye Pharma Group Ltd., Yantai, Shandong 264003, China; 3 School of Pharmaceutical Sciences and Institute of Material Medical, Binzhou Medical University, Yantai, Shandong 264005, China; 4 School of Pharmacy, Yantai University, Yantai, Shandong 264005, China; Columbia University, UNITED STATES

## Abstract

PFKFB3 (6-phosphofructo-2-kinase) synthesizes fructose 2,6-bisphosphate (F2,6P_2_), which is an allosteric activator of 6-phosphofructo-1-kinase (PFK-1), the rate-limiting enzyme of glycolysis. Overexpression of the PFKFB3 enzyme leads to high glycolytic metabolism, which is required for cancer cells to survive in the harsh tumor microenvironment. The objective of this study was to investigate the antitumor activity of PFK15 (1-(4-pyridinyl)-3-(2-quinolinyl)-2-propen-1-one), a small molecule inhibitor of PFKFB3, against gastric cancer and to explore its potential mechanisms. The effects of PFK15 on proliferation, apoptosis and cell cycle progression in gastric cancer cells were evaluated by cytotoxicity and apoptosis assays, flow cytometry, and western blotting. In addition, the invasion inhibition effects of PFK15 were measured by transwell invasion assay and western blot analysis, and a xenograft tumor model was used to verify the therapeutic effect of PFK15 *in vivo*. Results showed that PFK15 inhibited the proliferation, caused cell cycle arrest in G0/G1 phase by blocking the Cyclin-CDKs/Rb/E2F signaling pathway, and induced apoptosis through mitochondria in gastric cancer cells. Tumor volume and weight were also significantly reduced upon intraperitoneal injection with PFK15 at 25 mg/kg. In addition, PFK15 inhibited the invasion of gastric cancer cells by downregulating focal adhesion kinase (FAK) expression and upregulating E-cadherin expression. Taken together, our findings indicate that PFK15 is a promising anticancer drug for treating gastric cancer.

## Introduction

Gastric cancer is one of the most common gastrointestinal tumors and is the third leading cause of cancer-related mortality worldwide [1]. Radical surgery is the only curative therapy for non-metastatic gastric adenocarcinoma. However, gastric cancer is usually diagnosed in the local advanced or metastatic stage, with a 5-year survival rate lower than 30% [2]. Cytotoxic therapies have reached a plateau of efficacy. Many new targeted therapies aimed at specific oncogenic signaling pathways in gastric cancer have been developed and tested in clinical trials, but have not yielded great results [3, 4]. Hanahan and Weinberg’s recent update on the hallmarks of cancer [5] identified the reprogramming of energy metabolism as a new emerging hallmark, and has emerged as a therapeutic target to treat human cancers. Thus, we predicted that suppression of aerobic glycolysis might be useful in gastric cancer therapy.

Cancer cells are prone to maintain an abnormally high rate of aerobic glycolysis, a phenomenon known as the Warburg effect [6]. PFKFB3 is a key regulator of high glycolytic flux in cancers by catalyzing the synthesis of F2,6P_2_ which allosterically activates PFK-1, the rate-limiting enzyme of glycolysis [7]. PFKFB3 protein levels are overexpressed in a wide variety of cancers, including breast, prostate, colon, astrocytoma, ovarian, pancreatic and gastric cancers, and its expression or activity has been found to be strongly correlated with the aggressiveness and poor prognosis of the cancer [7, 8]. In addition, PFKFB3 has been reported to be associated with multiple oncogenic signaling pathways. For example, it has been reported that PFKFB3 is downregulated in cells with elevated phosphatase and tensin homolog (PTEN) [9], that PFKFB3 is phosphorylated on Ser^461^ downstream of the Sonic hedgehog (Shh) and AMPK signaling pathway [10], and that PFKFB3 is a direct transcriptional target of HIF-1α [11]. Given that PFKFB3 has been reported to be regulated by critical cancer-associated signaling molecules, strategies to inhibit PFKFB3 activity may be successful in suppressing cancer cell survival.

Inhibitors of PFKFB3 have been developed, such as 3-(3-pyridinyl)-1-(4-pyridinyl)-2-propen-1-one (3-PO) [12], N4A and YN1 [13], but their pharmacological activities cannot be solely ascribed to the inhibition of PFKFB3. Therefore, the development of a high selective inhibitor of PFKFB3, with low general toxicity inhibitor, is necessary. PFK15, a small molecular PFKFB3 inhibitor, displayed potent and selective activity against PFKFB3 and displayed antitumor capacity in various animal models [14]. However, there have been few reports about the effects of PFK15 against gastric cancer. In the present study, we demonstrate that PFK15 has on-target metabolic effects and antitumor activity in a gastric cancer model. We also investigated the possible mechanism of action of PFK15 in gastric cancer. Taken together, we believe that the antiglycolytic compound PFK15 is a promising candidate as an anti-cancer drug in gastric cancer therapy.

## Materials and Methods

### Animals and maintenance

Animals were housed and maintained in laminar-flow cabinets under specific pathogen-free conditions in a 12:12 h light-dark cycle and had free access to food and water. Temperature and humidity were maintained at 24±2°C and 50±5%, respectively. All animal researches were carried out in strict accordance with the National Institute of Health Guide for the Care and Use of Laboratory Animals (NIH Publications no. 80–23). Animal protocols were approved by the Ethics Committee of Shenyang Pharmaceutical University (No. 015 in 2013 for Animal Ethics Approval). Animal general status observation was monitored every day during the experiment. Animals were sacrificed after anesthetized and all efforts were made to minimize animal suffering.

### Reagents and chemicals

PFK15 (1-(4-pyridinyl)-3-(2-quinolinyl)-2-Propen-1-one) was purchased from Selleckchem (Houston, USA). Anti-E2F-1, anti-CDK (cyclin dependent kinase) 4 (DCS156), anti-cyclin E1 (HE12), anti-CDK6 (DSS83), anti-Rb (4H1), anti-CDK2 (78B2), anti-cyclin D1 (DCS6), anti-Bcl-2, anti-Bax (D2E11), anti-caspase 9 (C9), anti-caspase 8 (D35G2), anti-caspase 3 (8G10), anti-cleaved caspase-3 (Asp175), anti-FAK, anti-phospho-FAK (Tyr 397) and anti-β-actin antibodies were obtained from Cell Signaling Technology (Beverly, MA), anti-phospho-cadherin E and anti-Rb (phosphor S807) antibody purchased from Abcam (Abcam, MA).

### Cell culture and PFK15 treatment

MKN45, AGS, BCG823 (human gastric cancer cell lines) and GES-1 (human gastric epithelial cell line) were all obtained from Cell Culture Center of the Institute of Basic Medical Sciences, Chinese Academy of Medical Sciences and were cultured in Roswell Park Memorial Institute (RPMI) 1640 medium supplemented with 10% FBS (Gibco, USA) in an incubator at 37°C with 5% CO_2_ in air.

PFK15 was dissolved as a 40 mmol/L stock solution in DMSO and diluted with RPMI 1640 medium (Gibco) for *in vitro* experiments. The DMSO concentration was kept below 0.05% in the cell cultures in order to keep no detectable effects on the cells [15, 16]. In addition, the drug was suspended in 0.5% carboxymethyl cellulose sodium (SCMC) for *in vivo* experiments.

### Cell viability assay

The cell viability assay was determined by trypan blue exclusion [17, 14]. Briefly, cells were seeded into 96-well plates and incubated with PFK15 or PFK15 and 1 mmol/L F2,6P_2_ (sigma) for the indicated experiments. For dose-dependent experiments, PFK15 was added in increasing concentrations (0–20 μmol/L) for 24 h. For time-dependent experiments, 10 μmol/L PFK15 was added and incubated for 12, 24, or 48 h. For rescue experiments, 10 μmol/L PFK15 and 1 mmol/L F2,6P_2_ was added and incubated for 24 h. Cells were then collected for trypan blue staining and viable cells were counted via a standard hemocytometer. The 50% inhibitory concentration (IC_50_) values were calculated versus untreated controls using the GraphPad Prism 5 (GraphPad Software, Inc., USA).

### F2,6P_2_ and glucose uptake measurements

Intracellular F2,6P_2_ concentration was determined following the method as previously described [18]. Briefly, after treatment with dedicated concentration of PFK15 for 12 h, cell extracts were incubated in the mixture contained 50 mmol/L Tris/HCl, 5 mmol/L MgC1_2_, 0.15 mmol/L NADH, 1 mmol/L F6P (fructose 6-phosphate), 10 units/liter PPi (pyrophosphate)-dependent PFK1, 0.45 kilounit/liter aldolase, 5 kilounit/liter triose-phosphate isomerase, and 1.7 kilounit/liter glycerol-3-phoshate dehydrogenase (Sigma). Then, absorbance (OD = 339 nm) changes were analyzed after 0.5 mmol/L pyrophosphate was added. The F2,6P_2_ content was finally calculated and normalized to total cellular protein.

Glucose uptake was determined using the Glucose Assay Kit (Sigma) following the manufacturer’s instructions. Briefly, MKN45 cells were plated in complete medium and treated with PFK15 (0–10 μmol/L) for 12 h. Cells were then washed and starved in serum-free medium for 12 h, followed by 2-Deoxyglucose incubation for 1 h. After treatment, cells were lysed and the absorbance was measured at 412 nm.

### Cell cycle analysis

Cells were cultured in six-well plates at 3×10^5^ per well and incubated with PFK15 at 5 and 9 μmol/L for 24 h to explore a dose dependent analysis. A time course analysis was conducted in which cells were exposed to PFK15 for 12 h, 24 h or 48 h under the same concentrations. Then, cells were harvested and fixed in ice-cold 70% ethanol at 4°C overnight. The fixed cells were washed and resuspended with ice-cold phosphate-buffered saline (PBS) and incubated with 800 μL propidium iodide (PI) solution at 50 μg/mL in the dark for 30 min at 37°C. The stained cells were analyzed by FACSCalibur flow cytometer (BD Biosciences, San Jose, CA, USA), and cell cycle distributions were calculated with FlowJo v10.1 (Oregon, USA).

### Apoptosis analysis

Cells were cultured in six-well plates at 3×10^5^ per well and incubated with PFK15 at 5 and 9 μmol/L for 24 h to explore a dose dependent analysis. A time course analysis (0-48h) was also conducted under the same concentrations. Harvested cells were washed with PBS and stained with Annexin V and Propidium Iodide (Beyotime, China). Fluorescence was measured using a FACSCalibur (BD Biosciences, San Jose, CA, USA) and analyzed using FlowJo v10.1 (Oregon, USA). Annexin V^+^/PI^+^ (late apoptotic) and Annexin V^+^/PI^-^ (early apoptotic) cells were quantified by the frequency of fluorescently labeled cells and statistical significance was assessed by the two-sample t-test (independent variable).

### Cell invasion assay

For the transwell cell invasion assay, about 1.0×10^5^ cells were cultured in 0.1 mL serum-free medium along with the indicated concentrations of PFK15 at the top of Matrigel-coated chambers (24-well insert, 8-μm pore size; Corning, USA). The lower chambers were filled with 0.6 mL of medium containing 10% FBS. After 12 h, the cells were fixed in 4% paraformaldehyde and stained with 0.5% crystal violet. After removing the noninvasive cells on the upper surface of the filter by a cotton swab, the stained cells were quantified under a microscope with 200× magnification (Olympus DP80) in six randomly selected fields.

### Western blot analysis

MKN45 or AGS cells were normally cultured and directly treated with dedicated concentrations of PFK15 for 24 h. Cells were washed with PBS and lysed with radioimmunoprecipitation assay (RIPA) buffer, and then, supernatant was collected followed by centrifugation at 12,000 rpm for 20 min. Cell extracts (40 μg) were subjected to SDS-PAGE, and transferred to a polyvinylidene difluoride (PVDF) membrane. Membranes were probed with anti-E2F-1, CDK4, cyclin E1, CDK6, Rb, P-Rb, CDK2, cyclin D1, Bcl-2, Bax, caspase 9, caspase 8, caspase 3, cleaved caspase-3, FAK, P-FAK, P-cadherin E antibody, and horseradish peroxidase-conjugated secondary antibodies, and then detected with an enhanced chemiluminescence system (GE Healthcare Life Sciences) according to the manufacturer’s protocol. The β-actin antibody was used as a loading control in western blots for protein normalization. The optical density was quantified by ImageJ software.

### Xenograft studies

Female Balb/c nu/nu nude mice (4–5 weeks old; purchased from Vital River Laboratory Animal Technology Co., Ltd) were used for *in vivo* experiments. MKN45 cells (5×10^6^ cells per mouse) were resuspended in serum-free medium with matrigel basement membrane matrix (BD Biosciences) at a 1:1 ratio, and then subcutaneously injected into the right flank of each animal (5 animals/group). After tumors grew to about 120 mm^3^ in size, mice were treated intraperitoneally every three days with PFK15 at a dose of 25 mg/kg or vehicle for 15 days. Tumor dimensions and body weights were recorded every three days. The tumor weight was calculated using the formula (*l*×(*w*)^2^)/2, where *l* was the longest dimension of the tumor and *w* was the width. The inhibition rate (IR) of tumor growth was calculated by the following formula: IR (%) = [(A-B)/A]×100, where A and B were the mean tumor weight in the vehicle control and treatment groups, respectively.

### Statistics

Data were expressed as mean ± SD and analyzed using one-way analysis of variance followed by Dunnett’s test. Statistical analysis was performed using SPSS 18.0 software, and P<0.05 was considered statistically significant.

## Results

### PFK15 inhibits cell viability, F2,6P_2_ production and glucose uptake in gastric cancer cells

**Fig 1:**
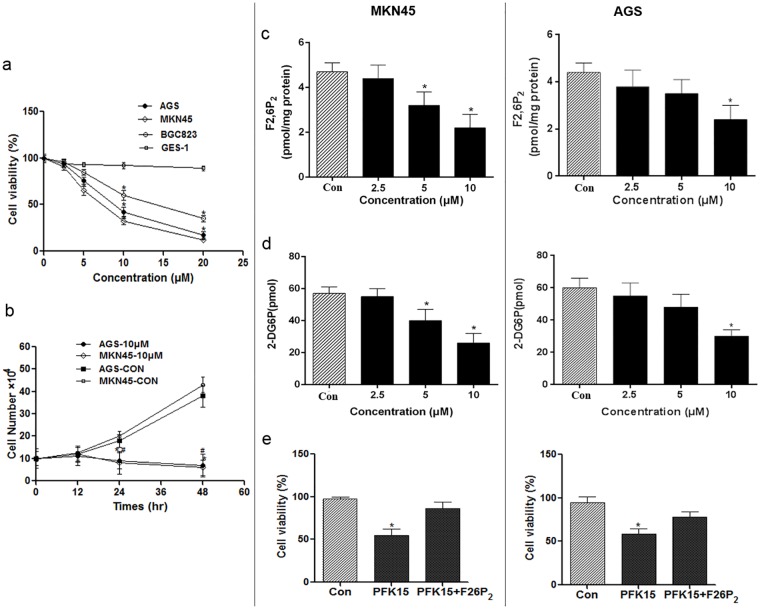
Effect of PFK15 on cell viability, F2,6P_2_ production and glucose uptake. (a) Cells grown in exponential phase were exposed to the indicated concentrations of PFK15 and, after 24 h, the effects on viability were determined by trypan blue exclusion. *p<0.05, compared with the GES-1 groups. (b) Time-dependent effects of PFK15 on MKN45 and AGS cell viability under the concentration of 10 μM. *p<0.05, compared with the control group of MKN45 cell line; ^#^p<0.05, compared with the control group of AGS cell line. (c,d) To identify acute effects on known metabolic effects of PFKFB3 inhibition, MKN45 and AGS cells were exposed to PFK15 for 12 h and the F2,6P_2_ and 2-Deoxyglucose uptake (Glucose Assay Kit) were measured. (e) The rescue effect of F2,6P_2_ on PFK15 induced cell proliferation suppression. The marked reduction of cell proliferation caused by 10 μmol/L of PFK15 in MKN45 or AGS cells could be partially rescued by F2,6P_2_ addition. Data are represented as the means ± SD from three independent experiments. *p<0.05, compared with controls.

PFK15 has been reported to be a specific and potent small molecule antagonist of PFKFB3, and is able to suppress the proliferation of various cancer cells at relatively low concentrations [14]. We examined the effects of PFK15 on gastric cancer cells by trypan blue exclusion and observed a dose-dependent suppression of cell proliferation after 24 h on MKN45, AGS, and BGC823 gastric cancer cells (IC_50_: MKN45, 6.59±3.1 μmol/L; AGS, 8.54±2.7 μmol/L; BGC, 10.56±2.4 μmol/L) and a time-dependent inhibition over 48 h on MKN45 and AGS cells under a concentration of 10 μmol/L (Fig 1a and 1b). Moreover, PFK15 caused <10% inhibition on normal gastric cells (GES-1) viability indicating that PFK15 is a selective inhibitor with low cytotoxicity in normal cells.

PFKFB3 is known to regulate high glycolytic flux in cancer cells [7]. Therefore, we examined the metabolic effects of PFK15 on the steady-state concentration of the substrate of PFKFB3, F2,6P_2_, as well as glucose uptake in MKN45 and AGS cells. The results indicated that PFK15 dose-dependently reduced F2,6P_2_ production and glucose consumption from 2.5 to 10 μmol/L in MKN45 and AGS cells respectively (Fig 1c and 1d).

Another F2,6P_2_ rescue experiment was performed and we found that the marked reduction of cell proliferation caused by 10 μmol/L of PFK15 in MKN45 or AGS cells could be partially rescued by F2,6P_2_ addition [Fig 1e, PFK15 mediated cell proliferation inhibition (% control): MKN45, PFK15 alone, 54.27±2.36%, PFK15+1mmol/L F2,6P_2_, 8.76±3.23%; AGS, PFK15 alone, 60.39±2.860%, PFK15+1mmol/L F2,6P_2_, 11.34 ±2.14%]. The observations further supported the specificity of PFK15 against the PFKFB3.

### PFK15 induces cell cycle arrest and alters the expression of key cell cycle proteins

**Fig 2:**
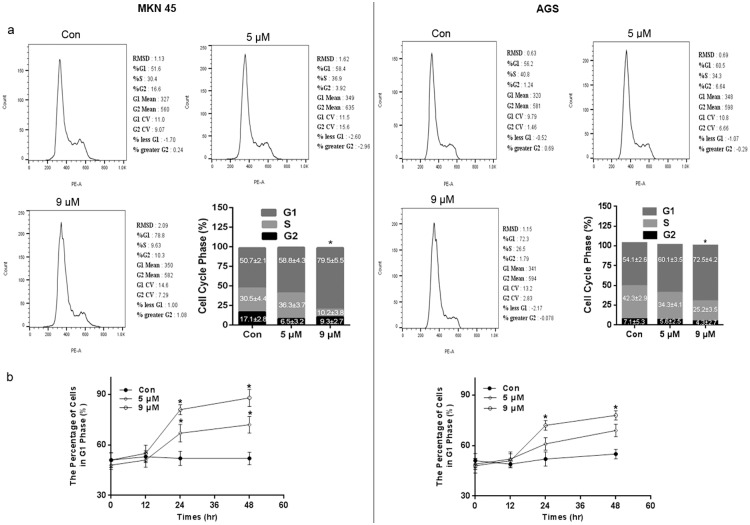
Role of PFK15 in cell cycle progression. (a) MKN45 and AGS cells were fixed and stained by PI following dedicated concentrations of PFK15 treatment after 24 h. Then the cell cycle distribution was analyzed using flow cytometer. PFK15 treatment remarkably prevented MKN45 cells from proceeding to S phase, thus resulting in cell cycle arrest at G0/G1 phase. (b) PFK15 treatment significantly increased the percentage of cells in G1 phase, indicating a time dependent effect on tested cells up to 48 h post-treatment. Data were analyzed by means ± SD and were representative of three independent experiments. *P<0.05, compared with controls.

PFKFB3 expression has been shown to be important for cell cycle progression [17, 19]. For example, PFKFB3-knockdown cells resulted in an increased percentage of cells in the S phase of the cell cycle [20]. Therefore, we investigated the role of PFK15 in the cell cycle changes in MKN45 and AGS cells using flow cytometry analysis, as described in methods. Treatment with PFK15 for 24 h caused cell cycle arrest at the G0/G1 phase in both cell lines (Fig 2a). The percent of cells in the G0/G1 phase significantly increased from 50.7% in control group to 79.5% (P<0.05) after 9 μmol/L of PFK15 treatment in MKN45 cells and 54.1% to 72.5% in AGS cells, respectively. Simultaneously, the proportion of cells in S phase decreased. A time related effect of PFK15 on cell cycle progression was also confirmed and a time-dependent increase in G1 phase cells was noted up to 48 h post-treatment (Fig 2b).

**Fig 3:**
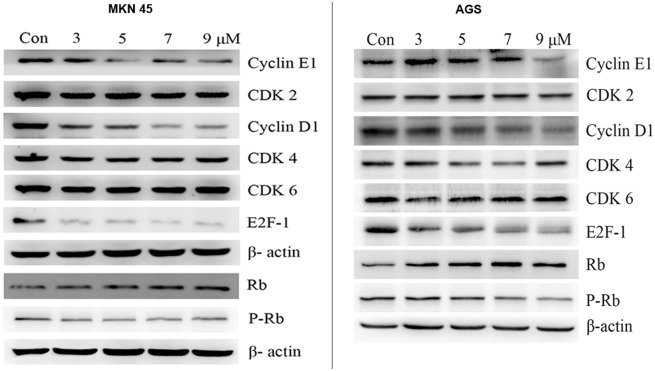
Mechanism of PFK15-induced cell cycle arrest in G0/G1 phase. Cells were treated with various concentration of PFK15 for 24 h. The cell cycle associated proteins were checked by western bolt assay. PFK15 treatment significantly decreased expressions of cyclin D1, cyclin E1, phosphorylated Rb and the nuclear transfactor E2F-1 levels while increased non-phosphorylated Rb protein levels in both MKN45 and AGS cell lines.

To examine the molecular mechanism responsible for the induced cell cycle block in G0/G1 phase by PFK15, the expression levels of G0/G1 regulatory proteins were determined. We observed that the expression levels of cyclin D1 and cyclin E1 were remarkably reduced by PFK15 after 24 h treatment at concentrations of 7 and 9 μmol/L in both cell lines (Fig 3). However, the compound had no effect on the protein levels of CDK 2, CDK 4 and CDK 6.

Retinoblastoma protein (Rb) regulates cell proliferation by controlling progression through the restriction point within the G1 phase of the cell cycle [21]. It also interacts with critical regulatory proteins including the E2F family of transcription factors. As shown in Fig 3, western blot revealed that E2F-1 and phosphorylated Rb expressions were dramatically downregulated by PFK15 treatment, and non-phosphorylated Rb was upregulated at the same dose in MKN45 and AGS cells, illustrating a transcriptional regulation of PFK15 in cell cycle block. All these data suggested that PFK15 could induce a G1/G0 block in gastric cancer cells by altering expressions of key proteins in the Cyclin-CDKs /Rb/E2F signal pathway.

### PFK15 causes apoptosis in gastric cancer cells through the mitochondrial pathway

**Fig 4:**
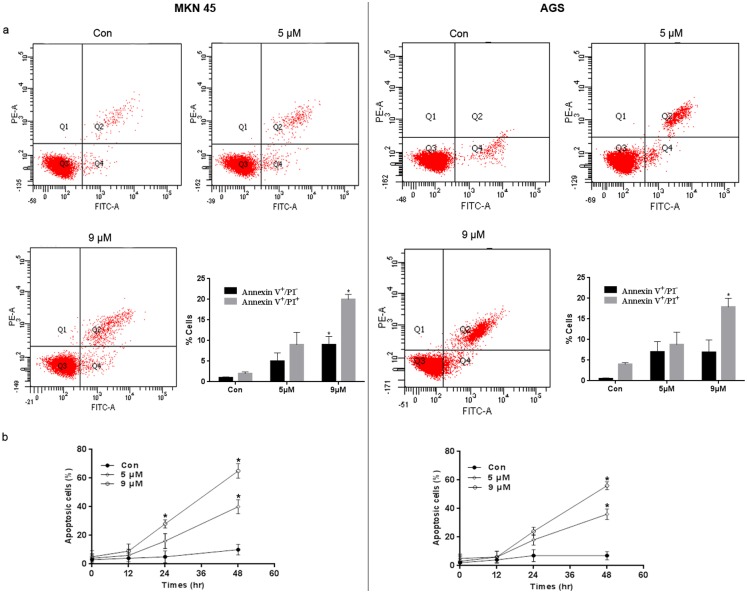
Role of PFK15 in cell apoptosis. **(a)** MKN45 and AGS cells were treated with various concentrations of PFK15 and collected after 24 h. Then cells were analyzed by flow cytometry after stained with Annexin V and PI. The percentages of early apoptotic (Annexin V^+^/ PI^-^) and late apoptotic (Annexin V^+^/ PI^+^) cells were quantified. (b) PFK15 treatment significantly increased the percentage of apoptotic cells, indicating a time dependent effect on tested cells up to 48 h post-treatment. Data were analyzed by means ± SD and were representative of three independent experiments. *P<0.05, compared with controls.

PFKFB3 expression is important for the maintenance of an anti-apoptotic state [17]. Consistent with these results, treatment with PFK15 resulted in apoptosis in MKN45 and AGS cells, as indicated by flow cytometry using an Annexin V-FITC Apoptosis Detection kit (Fig 4a). A time related effect of PFK15 on cell apoptosis was also confirmed and a time-dependent increase in apoptotic cells (early and late apoptotic cells) was noted up to 48 h post-treatment (Fig 4b).

**Fig 5:**
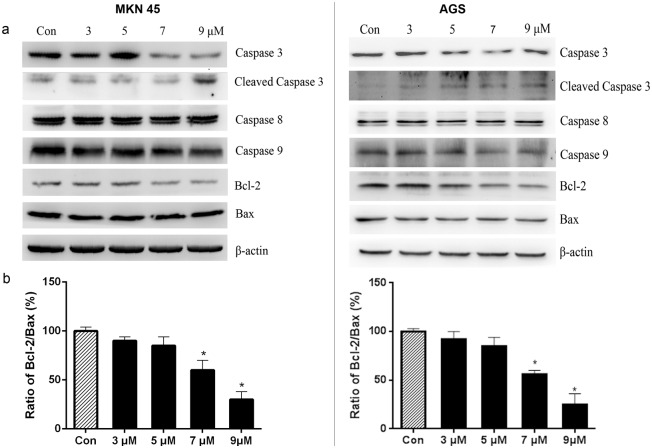
Mechanism of PFK15 induced apoptosis. (a) After treated with various concentrations of PFK15 for 24 h, MKN45 and AGS cells were collected and the protein changes were analyzed by western blot assay. (b) The optical density of Bcl-2 and Bax protein levels were quantified to controls by ImageJ software. PFK15 decreased the ratio of Bcl-2/Bax in a concentration-dependent manner. Columns, mean; bars, SD. *P<0.05, compared with controls.

The involvement of apoptosis was further confirmed by changes in apoptosis associated proteins after PFK15 treatment. For example, caspase 3 and caspase 9 protein levels were downregulated at concentrations of 7 and 9 μmol/L of PFK15, whereas caspase 8 protein levels were unchanged in both MKN45 and AGS cells (Fig 5a). In addition, cleaved caspase-3 protein expressions were significantly increased by PFK15 at 9 μmol/L in both cell lines.

Bcl-2 family proteins function different in the regulation of cell apoptosis and primarily affect the mitochondrial pathway [22, 23]. Bcl-2 stabilizes mitochondrial membrane and prevents the release of cytochrome c and other pro-apoptotic factors, whereas Bax promotes apoptosis. The ratio of Bcl-2/Bax is usually regarded as a criterion for programmed cell death [24]. PFK15 reduced the expression of Bcl-2 levels in the treatment groups, but had no effect on Bax expression levels in both gastric cancer cell lines (Fig 5a). Thus, the ratio of Bcl-2/Bax decreased in a concentration-dependent manner (Fig 5b). These results suggested that PFK15 induced apoptosis through the mitochondrial pathway by downregulating the protein levels of caspase 9, caspase 3 and Bcl-2, rather than the caspase 8 mediated extrinsic apoptosis pathway.

### PFK15 inhibits invasion of MKN45 and AGS cells

**Fig 6:**
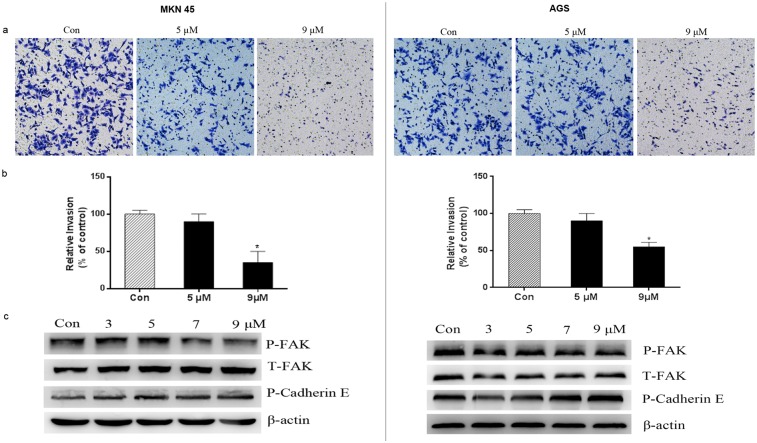
PFK15 inhibited the invasion of MKN45 and AGS cells. (a, b) Cells were treated with the indicated concentrations of PFK15 for 12 h in the upper chambers and the lower chambers were filled with 10% FBS medium. Then, the invaded cells were stained with crystal violet and observed under a microscope with 200× magnification. Data were obtained from six randomly chosen fields and were normalized to the control group. (c) Proteins related with cell migration and invasion were detected. Phospho-FAK levels were downregulated in 7 and 9 μM while phospho-Cadherin E levels upregulated in both cell lines. Columns, mean; bars, SD. *P<0.05, compared with controls.

Metastatic complications are responsible for more than 90% of cancer-related deaths, and cancer metastasis are one of the major obstacles in tumor therapy [25]. Previous studies have demonstrated the anti-metastasic effects of glycolytic inhibition in tumor cells accompanied by cytoskeletal rearrangements [26, 27]. Therefore, a modified Boyden chamber assay was performed to confirm the anti-metastasis properties of PFK15 on gastric cancer cells. Fig 6a and 6b demonstrated that a large number of cells invaded to the bottom layer of the membrane in the Boyden chamber in the control group, but the number of invading cells was significantly reduced by PFK15 treatment at 9 μmol/L concentrations in both cell lines.

We further investigated the role of PFK15 on cell invasion related proteins. PFK15 treatment decreased phospho-FAK expression levels at 7 and 9 μmol/L, and simultaneously increased phosphorylation of E-cadherin expression (Fig 6c). The results indicated that PFK15 treatment could inhibit the invasiveness of MKN45 and AGS cells, suggesting that it could potentially prevent cells from metastasizing.

### PFK15 inhibits MKN45 tumor growth *in vivo*

**Fig 7:**
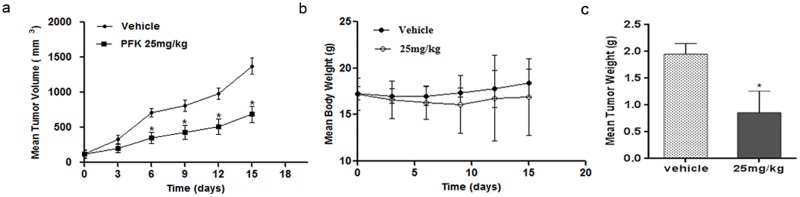
PFK15 suppressed tumor growth in gastric tumor xenograft models. MKN45 tumor-bearing mice were treated with PFK15 intraperitoneally at 25 mg/kg or vehicle every three days for 15 days. PFK15 had satisfactory inhibition effects against MKN45 tumor growth with an IR of 56.10% compared with vehicle group (a). Tumor volumes and body weight were measured twice a week (b, c). Columns, mean; bars, SD. *p<0.05, compared with vehicle controls.

To investigate the *in vivo* effects of PFK15 on gastric tumor growth we intraperitoneally injected mice bearing 120 mm^3^ tumors at 25 mg/kg or vehicle (SCMC) every three days for 15 days, according to Clem’s report [14]. PFK15 significantly inhibited tumor volume and tumor weight (IR, 56.10%) in a MKN45 xenograft model without a major effect on body mass (Fig 7a, 7b and 7c). Moreover, all the animals survived at the end of the treatment, suggesting limited toxicity. These data suggested that PFK15 could effectively suppress gastric tumor growth with no obvious side effects.

## Discussion

Gastric cancer is one of the most frequent cancers of the digestive system and has a high mortality rate worldwide. Currently, the first-line standard for treating gastric cancer is a cisplatin-based regimen of systemic chemotherapy, but unwanted side effects and variable treatment responses between individual patients has contributed to poor response rates. Targeted therapies aimed at specific cancer biomarkers have led to recent improvements in drug response and preventing drug resistance. However, disappointing results were achieved in most phase III clinical trials [28, 4, 3], and to date, only two drugs have been licensed. Trastuzumab was the first targeted agent approved as a first line therapy for HER2 positive patients, whereas ramucirumab was approved as a second-line treatment for patients with metastatic gastric cancer [29]. Furthermore, there remains a lack of validated clinically applicable biomarkers available for translation into routine clinical use [30]. Considering that deregulating cellular energetics is a new hallmark of cancer and aerobic glycolysis inhibitors have emerged as a promising therapeutic target [5], anti-glycolysis therapy may offer a new approach in gastric cancer treatment.

In this study, we investigated the antitumor activities of PFK15, a selective PFKFB3 inhibitor, and explored its potential mechanism of action in gastric cancer. The on-target metabolic effects of PFK15 were first confirmed in gastric cancer cells. We found that PFK15 treatment dose-dependently reduced F2,6P_2_ levels and glucose uptake in two gastric cancer cell lines, which was consistent with the anti-glycolytic effects reported in a previous study [14]. PFK15 had limited inhibition effects on normal gastric epithelial cells which indicated a selective inhibitor with low cytotoxicity. Another rescue experiment that F2,6P_2_ addition abrogated the PFK15-induced cell proliferation suppression in gastric cancer cells further supported the specificity of PFK15 for PFKFB3 enzyme. In addition to the significant suppression of cell proliferation in several gastric cancer cells by PFK15 treatment, a xenograft model was used to evaluate the general *in vivo* antitumor activity. The results demonstrated that PFK15 inhibited tumor volume and tumor weight, compared with the control. Besides that, results showing that no obvious adverse effects on body weight loss and no mortality indicated a well-tolerated profile of PFK15.

Based on *in vitro* and *in vivo* antitumor activity of PFK15 in our experiments, we further conducted experiments to understand the possible mechanisms of action of PFK15 in gastric cancer cells. We showed that PFK15 caused cell cycle arrest at G0/G1 phase in gastric cancer cells by altering the expression of various proteins involved in G1/S transition. PFK15 remarkably prevented MKN45 and AGS cells from proceeding to S phase, resulting in a much lower proliferative rate compared with control cells as indicated by flow cytometry. Then, we further demonstrated that the cell cycle arrest effects of PFK15 were associated with blocking the Cyclin-CDKs/Rb/E2F signal pathway. Rb acts to constrain the G1/S transition in mammalian cells. Cyclin D-CDK4/6 and Cyclin E-CDK2 complexes cooperatively contribute to the phosphorylation and inactivation of Rb [31, 32]. Phosphorylated Rb modulates E2F activation which is necessary for progression into late G1 and S phase [33]. This sequential modification provides additional specificity in regulating alternative cell fates such as proliferation and differentiation, and plays a critical role in tumor progression [32, 34]. PFK15 treatment downregulated cyclin D1, cyclin E1, E2F-1 and phosphorylated Rb levels, but upregulated non-phosphorylated Rb protein levels. Our results suggested that PFK15-induced cell cycle block was dependent on inhibition of Cyclin-CDKs /Rb/E2F signal pathway.

In addition to inhibition of cell cycle progression, PFK15 also had an apoptotic effect on gastric cancer cells indicated by Annexin V and PI staining. Furthermore, we found that PFK15-induced apoptosis may be dependent on the decreased ratio of Bcl-2/Bax, as well as the upregulation of cleaved caspase 3. Apoptosis can be initiated by activation of caspase 8 through the extrinsic pathway or caspase 9 through the intrinsic pathway. According to the western blot test results, PFK15 treatment had no effect on caspase 8 expression levels, but altered caspase 9 and Bcl-2 protein levels. In our study, these data suggested that PFK15 induced apoptosis through the intrinsic pathway involving mitochondria.

Tumor cells often invade surrounding tissues via blood and lymphatic vessels and form distant secondary tumors, which are critical for cancer prognosis. FAK is an intracellular cytoskeletal protein, and plays a significant role in various signaling pathways promoting cancer growth and metastasis such as cell motility, invasion and transcriptional events promoting epithelial-mesenchymal transition [35–37]. Moreover, E-cadherin is a critical member of the cadherin family that regulates cell adhesion, cell invasion and metastasis. It has been reported that phosphorylation of E-cadherin significantly enhances formation of the E-cadherin/β-catenin complexes and increases intercellular adhesion [38, 39]. In this study, we found that PFK15 was able to inhibit MKN45 and AGS gastric cancer cell invasion using an *in vitro* transwell invasion assay. In addition, we found that PFK15 treatment caused a decrease in phosphorylated FAK and an increase in phosphorylated E-cadherin protein levels. Our data suggested that PFK15 inhibited gastric cancer cell invasion, and this was partially dependent on the changes in FAK and E-cadherin expressions.

In conclusion, we confirmed that PFK15 exhibited anti-glycolytic effect in gastric cancer cells and further demonstrated that the antitumor activities of PFK15 were associated with cell apoptosis, cell cycle block and cell invasion inhibition in gastric cancer models. Our findings demonstrated that PFK15 is a promising anti-cancer drug for treating gastric cancer by targeting aerobic glycolysis and deserves further investigation.
